# 

**Published:** 1986-08

**Authors:** 


**Contents**

77 **No fault compensation for accidental injury**
Gordon M. Stirrat
79 **The History of Mental Handicap in Bristol and Bath (Part 2)**
J. Jancar
82 **Diabetes Mellitus and Mental Handicap—a preliminary report**
J. Jancar
83 **Upper gastro-intestinal endoscopy in a general surgical unit**
Colin D. Johnson
85 **From our Correspondents**
87 **Letter to the Editor**
88 **The Green Paper. An hypothesis to explain the delay in its appearance**
Alick Dowling
90 **Obituary—Dr Michael Lennard**
91 **An evening with Iris Murdoch**
92 **Book Reviews**
94 **South West Orthopoedic Club**
95 **On a bicycle made for two**
Alf Troughton, Rick Taylor

